# New developments in the pharmacotherapy of neuropathic chronic pelvic pain

**DOI:** 10.4155/fsoa-2016-0048

**Published:** 2016-10-05

**Authors:** Erin T Carey, Sawsan As-Sanie

**Affiliations:** 1 Division of Minimally Invasive Gynecologic Surgery, Department of Obstetrics & Gynecology, University of North Carolina School of Medicine, Chapel Hill, NC 27599-7570, USA; 2 Division of Minimally Invasive Gynecologic Surgery, Department of Obstetrics & Gynecology, University of Michigan Medical School, Ann Arbor, MI 48109-5624, USA

**Keywords:** chronic pelvic pain, drug development, medical management, neuropathic pelvic pain

## Abstract

Advancements in further understanding the pathophysiology of chronic pelvic pain syndromes continue to direct therapy. The mechanisms of chronic pelvic pain are often multifactorial and therefore require a multidisciplinary approach. The final treatment plan is often an accumulation of organ-specific treatment and chronic pain medications directed to the CNS and PNS. This article is a review of commonly used medications for chronic pelvic neuropathic pain disorders as well as an introduction to recent innovative developments in pain medicine.

## Lay abstract

Chronic nonmalignant pain, regardless of the cause, is a difficult disease to treat. While currently managed with a variety of medications, few alone are clinically effective. Often, many medications and therapies are used in combination to provide the maximum benefit for the patient. Several new medications listed below are being developed and applied to chronic pelvic pain.

Chronic pelvic pain (CPP) is a common gynecologic complaint. It is defined as noncyclic, nonmenstrual pelvic pain persisting >6 months, restricted to below the umbilicus [1]. It affects approximately 15% of women in the USA and is responsible for up to 20% of gynecologic office visits and 15% of hysterectomies; therefore it is not surprising that CPP is estimated to cost the healthcare system nearly US$2 billion per year [2–4].

The current evaluation and treatment of CPP includes a multidisciplinary approach secondary to the multifactorial nature of CPP. There is rarely a single identifiable cause and, despite the fact it is most likely to occur in women of reproductive age, it is estimated that only 30% of etiologies attributed to the development of CPP are gynecologic [5]. Pelvic pain can be further categorized as gynecologic, gastrointestinal, urologic, neurologic or musculoskeletal, though it is likely to result in the dysfunction of several organ systems (Table 1). As CPP is considered a chronic pain disorder (often with and without the presence of pelvic pathology), this review will evaluate current and potential future management of generalized chronic pain.

## Pelvic innervation

The management of CPP is most effective when a multifactorial approach is performed, in part due to the complex innervation of the pelvis, with a high rate of combined somatic (T12–S5) and visceral (T10–S5) pathology. The uterus, bladder and rectum are innervated by the hypogastric plexus, with sensory axons converging at the same dorsal root ganglion T10–L1. The vagina, clitoris and vulva as well as parts of the bladder, cervix and rectum also have sensory input from the sacral nerves (S2–S4) and also share sensory processing in the dorsal root ganglion of S2–S4 [6,7]. These communal information centers increase the risk of neuronal dysfunction in the pelvis, resulting in a unique cross-over effect. The sensitization of neighboring structures results in dysfunctional responses from the unaffected organs. This common effect, termed cross-sensitization, has been well described [8].

## Neurobiology of pain

While most chronic pain conditions are classified according to the mechanism by which they are thought to cause pain, this is often a misnomer (nociceptive vs neuropathic). Nociceptive pain is defined as pain arising from peripheral tissue inflammation or mechanical damage, from either somatic or visceral structures, and most often associated with acute pain complaints [9]. Nociceptive injury results in the subsequent release of pain modulating substances that stimulate afferent nociceptive fibers [10]. The release of substance P and gross mast cell activation results in neurogenic inflammation [11]. Common examples of nociceptive pain include postoperative pain and cancer pain [12]. Neuropathic pain, on the other hand, is pain derived from a lesion or dysfunction within the nervous system itself (e.g., peripheral neuropathy, herpes zoster), and it rarely has nociceptive stimulation [13].

However the above-described responses rarely follow their rigorous definitions in CPP. For example, endometriosis, long thought to be a solely nociceptive pain condition, has had a burst of recent evidence focused solely on the multifaceted neural mechanisms involved in the development and maintenance of endometriosis-related pelvic pain, beyond the simple pain at the site of the lesion [14]. This complex pain response explains why women may have persistent pain following excision of disease or hysterectomy and the well established finding that the stage of disease does not correlate with pain severity or intensity [14–16].

To further complicate CPP, a continuous pain signal from the pelvis may result in malfunctions of the neural pain response. This ‘malfunction’, known as sensitization, intensifies the pain signal from the periphery or its interpretation within the CNS. It is the abnormal amplification in pain processing which distinguishes acute from chronic pain.

Peripheral sensitization occurs from increased sensitivity at the level of the peripheral nerve due to a continuous nociceptive response [12]. This reduces the nerve activation threshold and makes them more reactive. Central sensitization, characterized by a CNS disturbance in pain processing, is thought to be an important in the pathology of many chronic pain syndromes (fibromyalgia, chronic low back pain, temporomandibular disorder and irritable bowel syndrome) [12]. In its purest form, there is no ongoing nociceptive stimulation from the periphery; however, there may be a combination of peripheral nociceptive signals and spontaneous signals from damaged peripheral nerves, resulting in neuronal remodeling along the pathway from the periphery to the pain processing center in the brain [12].

The mechanisms contributing to pain amplification and chronicity are heterogeneous and occur at multiple levels in the nervous system, including the brain. The complex composite of the pelvic pain pathway may explain the therapeutic response to central acting medications and neuromodulation techniques. Recent neuroimaging techniques have allowed the detailed description of the brain with functional MRI in women with CPP. In CPP, as in any other chronic pain state, there are many individuals with evidence of significant peripheral pathology with no pain, and others with severe pain who have little or no identifiable pathology. A critical construct is that within any diagnostic category (e.g., CPP), individual patients may have markedly different nociceptive, peripheral and central neural contributions to pain, and the balance between these factors determines the intensity of clinical pain and whether a patient responds to a given treatment. For example, women with CPP, regardless of endometriosis stage, are more likely to display hyperalgesia to experimental pain testing at a nonpelvic site [17]. Neuroimaging studies, which may reveal the neurobiological mechanisms of widespread hyperalgesia and altered pain sensitivity, have shown alterations in regional gray matter volume (GMV), chemistry and regional connectivity in CPP states [18].

Similar to fibromyalgia, decreased GMV in key pain regulatory regions such as the thalamus, cingulate gyrus, putamen and insula, as well as increased concentrations of excitatory neurotransmitters in the insula, have been shown in women with CPP (with and without endometriosis) [18]. Additionally, women with endometriosis without CPP did not have either hyperalgesia with pain testing nor changes in regional GMV. Instead, they exhibited increased GMV in the periaqueductal gray, a key structure in the endogenous pain inhibitory system. These data suggest alterations in brain physiology may be specific to the chronic pain state, rather than simply arising in anyone with peripheral pathology. Comparable changes in GMV have also been identified in women with cyclic pelvic pain without an associated peripheral pathology (primary dysmenorrhea) [19].

## Current medical treatment of chronic pain

As with most chronic pain syndromes, CPP rarely has a single source of pain generator, and often presents with a blend of neuropathic pain, a continuous inflammatory response and secondary muscle dysfunction. Unfortunately, the current medical regimens have resulted in less than optimal outcomes for pain management; in part, due to the pathogenic heterogeneity found in CPP. A recent Cochrane review for the medical treatment of CPP limited the medical interventions to anti-inflammatories, hormonal treatment, anticonvulsants, anticholinergics, antidepressants and local anesthetic injections [20]. CPP can be a combination of a visceral and neuropathic pain component, and in these cases effective medical therapy is often achieved with a combination of medications working in the periphery as well as on the CNS. The current approach to CPP is generally a classic approach of trial and error, discontinuing one medication before beginning another. In many instances, multimodal drug therapy can be effective by targeting different pain pathways. Vulvodynia, chronic pain limited to the vulva and organ-specific pain disorders (endometriosis, interstitial cystitis, irritable bowel syndrome) have not been included in this review, though there may be an underlying neuropathic pain disorder to all of these syndromes.

## Analgesics

Regardless of the etiology of the pain, traditional analgesics play an important role in the management of CPP. These are effective for acute pain ‘flares’ as well as maintenance therapy, and may be used alone or in combination: aspirin, nonsteroidal anti-inflammatory drugs (NSAIDs), paracetamol (acetaminophen), and opioids. NSAIDs work by nonselective inhibition of the cyclooxygenase (COX) enzyme preventing the production of prostaglandins and thromboxane. The inflammatory blockade can be effective in the peripheral pain response. Much of the research in the efficacy of anti-inflammatories and CPP are founded in the physiology of the uterine environment through menses. In the late luteal phase, decreased progesterone results in increased arachidonic acid production, metabolized by the COX-2 pathway into eicosanoids [21]. Eicosanoids have been considered a main contributor to the inflammatory pain in dysmenorrhea, and they are known to substantially decrease with NSAID treatment [22]. Selective COX-2 inhibitors have also been successfully used in the management of primary dysmenorrhea, endometriosis and CPP specifically, though the risk of scheduled use of NSAIDS remains high with effects on the renal, hepatic, gastrointestinal and cardiovascular system [23]. A systematic review by Latthe *et al*. identified that NSAIDs can reduce moderate and severe pain associated with dysmenorrhea when compared with placebo [24]. A recent Cochrane review confirmed that NSAIDs were highly efficacious in the treatment of dysmenorrhea, though frequency of use may be limited by side effects [25]. While aspirin also inhibits the COX enzyme, and its analgesic effects are secondary to a reduction in inflammation [26], few studies have shown significant improvement in pain associated with menses when compared with placebo [24].

The exact mechanism of action of paracetamol is unknown, however, it is also believed to work by inhibiting central prostaglandin synthesis, and works well as a drug potentiator, increasing the effectiveness of other analgesic medications. Dose-related hepatotxicity is a major known risk of acetaminophen [27]. Few studies have identified it as an effective pain reliever alone [24]; however, when combined with NSAIDs or caffeine, paracetamol has been shown to achieve moderate levels of pain relief with menstrual pelvic pain [28,29].

Opioids are highly effective for acute pain and chronic malignant pain; however, their role in chronic nonmalignant pelvic pain remains controversial. In fact, there is extremely limited data on the role of opioid therapy and pelvic pain [30], opioid receptors are G-protein receptors with three known subtypes μ, δ and κ and these receptors are primarily located in the brain (cortex, thalamus and periaqueductal gray) and spinal cord. Traditionally, analgesics have directed therapy to the μ receptor or the δ receptor (though activation of the latter is responsible for the significant side effect profile that can accompany these medications despite analgesic effect) [31]. Opioid receptors are found in both the CNS and PNS and the gastrointestinal system. While excellent analgesics, the short and long-term side effect profiles remain high, and the risks of long-term opioid use should be seriously measured in reproductive aged women with CPP.

## Antidepressants

While antidepressants are widely used in the treatment of chronic pain syndromes, there is limited evidence of their use in women with CPP [20]. Tricyclic antidepressants (TCAs) are a first-line treatment of many neuropathic chronic pain conditions, increasing the amount of available norepinephrine, thus reducing pain [32]. Unfortunately, data pertaining to CPP in women are minimal. One study randomized 56 women with CPP to amitriptyline, gabapentin to amitriptyline/gabapentin combined for 24 months. While each drug and drug combination resulted in a significantly reduced pain response, fewer side effects were noted in gabapentin alone when compared with the addition of amitriptyline [33]. Unfortunately, poor compliance and early discontinuation is common due to the anticholinergic side effects [34]. Nortriptyline and imipramine have also been studied limited in small groups of women with CPP with some improvement of pain symptoms [35,36]. The majority of the studies evaluating TCAs effect on pelvic pain is restricted to urologic pain disorders, not necessarily generalizable to women with CPP without urologic symptoms [37–40].

Serotonin is an important neurotransmitter in the development and maintenance of depression and anxiety. It also is a modulator of the descending inhibitory pain pathways in the CNS. Increasing the availability of serotonin may affect pain disorders. The selective serotonin reuptake inhibitors sertraline, has been studied in a single, small, placebo controlled randomized controlled trial in women with CPP, in which 23 women were randomized to sertraline or placebo. Despite 6 weeks of use, there was no notable difference in pelvic pain scores between the groups [41].

Like serotonin, norepinephrine also inhibits pain by inhibiting the descending pain pathways. Selective neurotransmitter reuptake inhibitors result in the increased availability of serotonin and norepinephrine, and have been highly successful in the treatment of many pain disorders. The significant analgesic effect may be predominately from the increase in norepinephrine centrally. While no studies have been performed in women with CPP exclusively, duloxetine has been identified as an effective pain modulator in urologic pelvic pain disorders in men and women [42,43] and is widely used in the treatment of diabetic peripheral neuropathy, fibromyalgia and chronic musculoskeletal pain [44].

Other antidepressants have shown modest improvement in chronic pain include bupropion (noradrenergic and dopaminergic pump inhibitor) and trazodone (serotonin-2 antagonist/reuptake inhibitor), though again there remains a lack of literature pertaining specifically to women with CPP.

## Membrane stabilizers

### Calcium channel blockers

Gabapentin and pregabalin, both calcium channel blockers, decrease the reuptake of glutamate, norepinephrine and substance P and operate as membrane stabilizers peripherally and centrally. While traditionally prescribed for neuropathic pain, these have been successfully employed in nonspecific pain conditions as well as those with primary musculoskeletal dysfunction (i.e., fibromyalgia) [12].

In women with CPP without known gynecologic pathology, gabapentin is the one of the medications with the most evidence of efficacy. Lewis *et al*. randomized 47 women to gabapentin treatment vs placebo for 6 months in a pilot study. Gabapentin dose was titrated until the patient met a 50% reduction in reported pain (maximum dose 2700 mg/day). In one measure of pain, there was improvement of pain in the treatment group [45]. No current published studies pertaining to the use of pregabalin and CPP in women exist; however, a large randomized controlled trial in men with chronic prostatitis/CPP syndrome (thought to be a neuropathic pain condition) did not show a significant improvement in pain symptoms after 6 weeks of use (up to 600 mg/day) when compared with placebo [46]. Pregabalin has been shown to be highly effective in other neuropathic pain disorders such as postherpetic neuralgia, diabetic neuropathy and fibromyalgia [47–49].

### Sodium channel blockers

Sodium channel blockers work by decreasing overall neuron membrane excitability and reduce the spontaneous firing of sensory neurons. Anticonvulsants such as phenytoin, carbamazepine, oxcarbazepine, lamotrigine, tigabine and topiramate have been studied and found to be effective in many neuropathic pain conditions [50]. The mechanism of action of local anesthetics is also by sodium channel blockade. No studies specific to chronic pain in the female pelvis have been identified with these medications.

## Progestins

Endometriosis is a common comorbid pain disorder in women who also have CPP, however, endometriosis-specific treatment will not be addressed in this review. While progestin therapy is an effective treatment option for endometriosis-related pain, it has also shown efficacy in CPP without the pathology of endometriosis [20]. Medroxyprogesterone acetate has been identified as a first-line treatment in women with pelvic pain secondary to pelvic congestion syndrome [51].

## Other

Several other medications are commonly used in the management of CPP, depending on the presumed etiology. Anxiolytics and typical/atypical neuroleptics have also been used with some benefit in the treatment of pain disorders. While some anxiolytics provide an analgesic effect through the increase of inhibitory GABA and subsequent reduction in motor neuron excitation, cautious use recommended due to risk of dependency and abuse [12]. There are no published studies specific to women with CPP.

## Emerging therapies

### Topicals

Capsaicin is a compound that selectively binds to the ion channel receptor, vanilloid receptor subtype 1 (TRPV1). This receptor is found almost exclusively on neurons that relay heat and pain and expression of these receptors have been identified as pain modulators in individuals with endometriosis and CPP [52,53]. The proposed mechanism of action is basic desensitization of the unmyelinated C nerve fibers found to contribute to some pelvic pain syndromes. In a small clinical trial of 22 male patients with chronic prostatitis/CPP symptoms (and six healthy controls), a single application of 5 ml of topical capsaicin was applied to the perineal skin. While the cases reported significantly more discomfort with the application of the cream, 16 of the 22 cases reported improvement of pain symptoms at 1 week following the initial application. Further studies are needed to evaluate the long-term efficacy of this use as a topical compound in CPP [54].

Built on the success of sodium channel blockers, directed voltage-gated sodium channel blockers are currently a focus of new drug development. Thus far, nine α-subunits (Nav1.1–1.9) and four β-subunits (β1–4) have been classified in humans. Nav1.7 has a specific interest to drug makers due to its peripheral activity and specificity to pain. While an inoperative Nav1.7 channel has been identified in persons with a congenital insensitivity to pain, other malfunctions of the same channel can results in hyperexcitability and rapid misfiring of the sodium channel, resulting in severe pain (paroxysmal extreme pain disorder). While many oral compounds have failed to advance to clinical trials, a topical compound created by Xenon Pharmaceuticals called XEN402 is undergoing clinical trials for herpetic neuralgia and may be applied to other pain disorders in the future [55].

### Toxins

Botulinum toxin A, produced by the bacterium *Clostridium botulinum*, is a powerful neurotoxin. It significantly inhibits the release of the neurotransmitter acetylcholine from nerve fibers, resulting in short-term paralysis [56]. This has been effective in treating pelvic pain disorders by the aborting persistent muscle contractions [57]. The first use in women with CPP was a small study of 12 women with pelvic floor hypertonicity in which 80 units of botulinum toxin A was injected into the levator muscles with significant reduction in resting pelvic floor pressure and dyspareunia at 4 weeks [58]. Subsequently, a randomized controlled trial was performed with 60 women randomized to botulinum or normal saline. In the placebo group there was a change in dyspareunia but not resting pressure [59]. In addition to the inhibition of acetylcholine and subsequent muscle contraction, it also blocks other neurotransmitters which regulate pain, including glutamate, substance P and calcitonin gene-related peptide and may contribute to its analgesic effect [60].

### Modified opioids

While opioids are well established in the management in moderate to severe pain, the risk and side effect profile as well as the inability to sufficiently provide pain relief in many patients leaves room for improvement. Tapentadol is a modified opioid, combining the analgesic effects of acting as a centrally acting μ-opioid agonist with a mild norepinephrine reuptake inhibitor. This allows a weaker affinity to the μ-opioid receptors than traditional opioids (which improve the side effect profile) while maintaining pain relief through dual mechanisms. The US FDA has approved tapentadol for chronic moderate to severe pain as well as the first opioid approved for the treatment of diabetic peripheral neuropathy [61].

Identifying new medications with combined μ agonistic and δ antagonistic effects appear to reduce the side effect profile and the development of tolerance when compare to currently available medications. One of these medications is a drug under development called UMB 425, as potent as morphine, with decreased tolerance and minimal toxicity [31]. Two drugs derived from this substance are currently undergoing Phase I clinical trials. Consistent with opioid therapy and CPP described earlier, no studies have been performed specifically evaluating the efficacy of modified opioid therapy in women with CPP.

### Cannabinoids

Cannabinoid receptors, cannabinoid 1 (CB1r) and cannabinoid 2 (CB2r), are G-protein-coupled receptors for endocannabinoids. The majority of the psychotogenic properties are caused by activation of CB1r as they are primarily found in the CNS, whereas CB2r are mainly found on monocytes and microglia. Cannabinoid ligands have also shown to have a role in nociception of pain. Due to this, many therapies have been directed toward CB2r agonists or cannabinoid ligands to avoid side effects in the CNS. Interestingly, CB1r have also been identified in the mouse urinary bladder and a reduction in bladder nerve activity has been identified when activated [62]. This finding correlates with a study in patients with painful bladder syndrome and idiopathic detrusor instability. Bladder biopsies revealed an increase in CB1r-immunoreactive nerve fibers in patients with bladder pain when compared with controls [63]. These findings are supportive of new development of selective CB1r agonists in the future.

The Canadian Pain Society recently included cannabinoids as third-line treatments for chronic neuropathic pain behind first-line agents such as gabapentinoids, TCAs and serotonin noradrenaline reuptake inhibitors, and second-line agents including tramadol and controlled-release opioids [64]. The role of cannabinoid receptor and ligand agonists will continue to evolve as directed therapy development, acceptance and availability increase in the USA. No studies have been performed or published in relation to the effect of pain management in women with CPP.

### N-methyl-d-aspartate glutamate receptor antagonists receptor blockers

N-methyl-d-aspartate (NMDA) glutamate receptor antagonists block the receptor at the neurotransmitter site. Ketamine is a NMDA used for both its anesthetic and dissociative properties and is currently being evaluated for a role as a rapid acting antidepressant, particularly in bipolar patients [65]. Memantine is also a NMDA receptor antagonist currently approved by the FDA for Alzheimer's disease, but its potential uses are diverse, including the treatment of chronic pain [65,66] obesity [67], depression [65,68–69] and schizophrenia [70]. Pre-emptive use of memantine in rats prior to spinal nerve ligation have also shown promise in inhibiting the development of neuropathic postoperative pain and may be a target of pre-emptive analgesia drug development [71]. As peripheral NMDA sites are also important components of visceral pain processing, targeted therapies may be directed in the future [66].

### NGF inhibition

NGF is a hormone released from mast that mediates pain transduction at the level of nociceptive neurons and is increased in the setting of inflammation. While necessary for neuronal differentiation during development, later it is responsible for generating pain in response to nociceptive injury. Tanezumab, a recombinant humanized monoclonal antibody targeting NGF-associated pain, is currently undergoing clinical trials for the treatment of chronic low back pain and osteoarthritis. A proof of concept study randomized 62 patients with chronic prostatitis/CPP syndrome to receive a single dose of tanezumab (30) or placebo (32) with minimal improvement in pain symptoms in this small group [72]. Despite these findings, it continues to be an appealing therapeutic target for future studies in chronic pain disorders, particularly diseases associated with a high inflammatory response such as endometriosis [73,74], though no published studies specific to CPP were identified.

## Conclusion

The management of CPP is often anecdotal, with few randomized clinical trials identifying effective single treatments of pelvic pain disorders. While there are several available consensus guidelines and expert opinions provide algorithms for specific pain disorders affecting the pelvis [1,75], in general recommended therapy remains multidisciplinary. Similar to other pain disorders, the addition of physical therapy, psychotherapy, optimization of lifestyle factors and complimentary medicine practices may improve pain outcomes when compared with medical management alone.

Novel drug developments targeting pain therapy continue to be of interest to pharmaceutical companies as well as modifying currently available drugs to improve efficacy and decrease side effects. While disease specific therapy, such as hormonally directed therapy of endometriosis and endometriotic lesions continue to propagate, the general management of chronic pain syndromes will continue to follow other pain disorders. Available medications may be used synergistically to improve pain outcomes. By no means does this article provide an all-inclusive list of new medications for pain, however it does describe some that may be readily incorporated in clinical practice in the near future. An obvious limiting factor to the identification and implementation of effective treatment interventions for many pelvic pain conditions is gross heterogeneity in the treatment groups [76]. Continued diligence in the strict definition of the neuropathic pelvic pain disorder will allow the identification of effective drugs that are currently being used as well as the appropriate evaluation of developing therapies.

## Future perspective

Many medications used currently are prescribed ‘off-label’ for pain management despite well-established analgesic efficacy. Novel targeted therapies specifically for chronic pain continue to emerge. Regardless, multimodal therapy will continue to lead the way for optimal pain management for CPP disorders.

**Table 1.** **System-based etiologies of chronic pelvic pain.**

**Organ system**	**Disease**
Gynecologic	Endometriosis, adenomyosis, ovarian remnant, pelvic congestion/pelvic venous insufficiency, pelvic inflammatory disease, ovarian cysts, uterine leiomyomas, tubal pathology (hydrosalpinx, pyosalpinx), adhesive disease
Neurologic	Nerve entrapment/irritations/impingement, disc herniation, postherpetic neuralgia, visceral sensitivity
Gastrointestinal	Irritable bowel syndrome, inflammatory bowel disease, chronic appendicitis
Urologic	Bladder pain syndrome/interstitial cystitis, urethritis
Musculoskeletal	Fibromyalgia, abdominal wall myalgias, pelvic floor tension myalgias, sacroiliac joint dysfunction, symphysis pubis pain, coccydynia
Psychological	Anxiety/depression, somatization disorders, psychosexual dysfunction, sexual abuse, post-traumatic stress disorder

**Executive summary**

Chronic pelvic pain is a clinical pain disorder affecting a significant number of reproductive aged women.
Despite the broad list of causes of chronic pelvic pain, once the pathology (if present) is treated, the disease of pain must also be addressed.
Current adopted treatments include analgesics, anesthetics, antidepressants and membrane stabilizers.
Novel treatment therapy includes new topical applications, the use of specific toxins with analgesic effects, modification of opioid medications and manipulation of neurotransmitters and hormones responsible for the mediation of pain.
Despite targeted drug development, patients will likely continue to receive the most benefit in the setting of multimodal drug therapy and a multidisciplinary approach.
